# Impact of Standardized Prenatal Clinical Training for Traditional Birth Attendants in Rural Guatemala

**DOI:** 10.3390/healthcare6020060

**Published:** 2018-06-09

**Authors:** Sasha Hernandez, Jessica Oliveira, Leah Jones, Juan Chumil, Taraneh Shirazian

**Affiliations:** 1 Saving Mothers, New York, NY 10022, USA; joliveira@savingmothers.org (J.O.); ljones@savingmothers.org (L.J.); tshirazian@savingmothers.org (T.S.); 2 Ministerio de Salud Pública y Asistencia Social, Santiago Atitlán, Sololá 07019, Guatemala; jchumilcuc@hotmail.com

**Keywords:** birth attendant, prenatal care, clinical skills, education, maternal mortality, indigenous health, rural, Guatemala

## Abstract

In low-and-middle-income countries (LMICs), traditional birth attendant (TBA) training programs are increasing, yet reports are limited on how those programs affect the prenatal clinical abilities of trained TBAs. This study aims to assess the impact of clinical training on TBAs before and after a maternal health-training program. A prospective observational study was conducted in rural Guatemala from March to December 2017. Thirteen participants conducted 116 prenatal home visits. Data acquisition occurred before any prenatal clinical training had occurred, at the completion of the 14-week training program, and at six months post program completion. The paired *t*-test and McNemar’s test was used and statistical analyses were performed with R Version 3.3.1. There was a statistically significant improvement in prenatal clinical skills before and after the completion of the training program. The mean percentage of prenatal skills done correctly before any training occurred was 25.8%, 62.3% at the completion of the training program (*p*-value = 0.0001), and 71.0% after six months of continued training (*p*-value = 0.034). This study highlights the feasibility of prenatal skill improvement through a standardized and continuous clinical training program for TBAs. The improvement of TBA prenatal clinical skills could benefit indigenous women in rural Guatemala and other LMICs.

## 1. Introduction

Significant worldwide progress has been made towards lowering rates of maternal mortality in the last two decades [1]. Yet maternal mortality ratios (MMR) in the developing world, especially in rural regions and within indigenous populations, continue to be unacceptably high [2]. This holds true in Guatemala where the national average of 88 maternal deaths per 100,000 births [1] does not reflect the major disparities that exist between the MMR for indigenous women which is twice that of their counterparts (163 per 100,000 compared to 78 per 100,000) [3]. In recent years, the Guatemalan government has aimed to decrease this discrepancy by promoting institutional births [4], however, up to 70% of indigenous women living in rural Guatemala continue to deliver at home without receiving adequate prenatal care [5]. This continued preference of home care during pregnancy and delivery is due both due to limited access to essential obstetric care [6] and a non-wavering cultural preference for traditional birth attendants (TBAs) to attend to women at home [7]. 

TBAs are important to both the local community and national health infrastructure as their training and integration in the current healthcare system can help improve maternal and neonatal outcomes [8,9,10,11]. Despite the significant role TBAs hold, there are limitations, as few sustainable training programs exist that properly train TBAs in how to provide basic prenatal care, detect early complications, or refer high-risk pregnancies in a standardized way. TBA participants themselves report that they are not always given sufficient training during these programs [12]. Even for successful programs, challenges exist in measuring Prenatal Clinical Skills (PCS) and reporting trends over time. While some studies have attempted to evaluate PCS, they have focused on simulated sessions [13] and cross-sectional views [14]. Such studies do not provide a direct look at how a training program affects the PCS of their trainees before and after training and how those skills develop after continued training. 

The School of POWHER, which stands for Providing Outreach in Women’s Health and Educational Resources, is a yearly TBA training program that has been successfully implemented in the rural communities of the department of Sololá, Guatemala over the last four years and supported by both community infrastructure and the regional branch of the Guatemalan Ministry of Health. The School of POWHER training program is an immersive program, delivered in both Spanish and Tz’tujil (a local Mayan language), based on a two-pronged approach. There is a 28-module lecture series (which runs over 14 weeks, with two four-hour lectures given per week) emphasizing signs of referrals for the mom and baby, prenatal care, and initial management of post-partum complications (Figure S1, see Supplementary Materials). A separate 12-month clinical constituent emphasizes basic prenatal home care and appropriate referral. The clinical arm of the school ensures that TBAs refine their traditional midwifery abilities while learning new skills under the supervision of a preceptor. During bi-monthly home visits in the students’ communities over twelve months, the TBAs will practice items, including but not limited to, counseling the mother about vaccines, measuring blood pressure, using a fetal Doppler, and correctly estimating delivery date. The School of POWHER respects cultural Mayan practices and its content is in line with current WHO and Guatemalan healthcare guidelines for TBAs [15,16]. Each woman that completes the School of POWHER training program receives a stethoscope, blood pressure equipment, a fetal Doppler, prenatal vitamins, and safe birthing kits. In total, the training program, now a one hundred percent sustainable program as current School of POWHER staff are TBAs who are past graduates of our program, has trained 60 TBAs in southwest Guatemala in focused maternal healthcare with appropriate obstetric referrals as the key to decreasing maternal death and complications in the region [17]. 

The main objective of the present study was to assess the effect of the School of POWHER’s clinical training on its participants after (1) completion of the program and (2) after six months of continued clinical training. The time of notable improvement of PCS, as well as level of adherence over time, was also studied. 

## 2. Materials and Methods

A community-based, prospective observational study was conducted in six rural communities throughout the department of Sololá, Guatemala from March through December 2017 to assess direct PCS acquisition and retention from the School of POWHER training program during prenatal home visits.

All TBAs recruited and then enrolled in the School of POWHER training program during the time of this study were included. TBA demographics are described in Table 1.

Data collection was scheduled at three different time points from March through December 2017 with the goal of capturing PCS during home visits prior to and after School of POWHER clinical training. Home visits were arranged with each TBA student in their respective communities with their existing patients. If a TBA student did not have current pregnant patients and/or a visit could not be scheduled with her patient, the Ministry of Health provided a list of pregnant women in each community to visit. Each time point and the corresponding amount of clinical training are defined in Table 2. The goal was to observe 1–5 prenatal home visits by each TBA student in their own community during each of the three time points. Two weeks were allotted after each time point to collect all observational data from the 13 TBA participants. During each two-week data collection time point, no additional clinical instruction occurred to ensure that each participant had received the same amount of clinical training. 

During each time point of the study period, prenatal home visits were observed with a WHO-adapted prenatal care checklist tested in our prior pilot study [18]. This WHO-adapted checklist covered three broad categories of medical history, clinical skills, and counseling (Figure S2, see Supplementary Materials). The medical history information included age, number of previous pregnancies, complications with previous pregnancies, other significant medical history, current medications, current weeks of gestation, and estimated date of delivery. The clinical skills evaluated included measuring the maternal blood pressure, fundal height, fetal heart rate, and fetal position. Counseling included reviewing signs and symptoms of danger during pregnancy and what course of action to take if danger symptoms presented. A review of the emergency birth plan, the importance of tetanus vaccination, and prenatal vitamins were also evaluated. All study variables and appropriate reasons for referrals are defined in Table 3.

On arrival at each home, the evaluator observed the prenatal home visit after patient verbal consent was obtained from the TBA conducting the visit. During the data collection process, one of two evaluators observed all prenatal home visits. These two evaluators were trained on the POWHER curriculum and observation checklist before the commencement of the study. Evaluator A, a senior medical student, was in charge of data collection during the pilot study period to Time Point A. Evaluator B, a medical volunteer with public health experience, observed the prenatal home visits from Time Point B to Time Point C. A translator assisted the evaluator for visits that occurred in native languages (Tz’utujil and Kaqchikel). After the completion of the observed visit, the evaluator had an opportunity to intervene if maternal or fetal referral was indicated, but missed by the TBA during the observation. This referral was not included as part of this study. No personal patient identifiers were collected to maintain the confidentiality of the women.

Data analysis was carried out using standard statistical methods. The paired *t*-test and McNemar’s test was used and statistical analyses were performed with R, Version 3.3.1 (R Core Team, Vienna, Austria). 

## 3. Results

**Figure 1:**
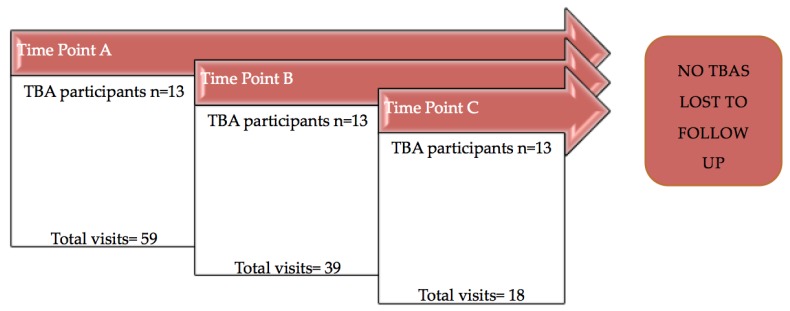
The total participants and observed visits over the study period.

A total of 116 prenatal home visits by 13 TBAs were observed. Each TBA participant was evaluated at each of the three time points as no participants were lost to follow-up. A total of 59 visits occurred at Time Point A, 39 visits occurred at Time Point B, and 18 visits occurred at Time Point C. For TBAs with multiple observed home visits during each time point, their results were averaged over their visits (Figure 1).

No study participants were lost to follow up from March to December of 2017. Total observed visits differed from each of the three time points due to the difficulty in reaching each rural community due to nationwide transportation strikes. The median number of home visits per TBA during time point A was 3 with a range of 1–9, 2 home visits per TBA during time point B with a range of 1–8, and 1 home visit per TBA during time point C with a range of 1–3.

The overall improvement in prenatal clinical skills is shown in Table 4. The mean percentage correct on the checklist before any training occurred was 25.8% (Time Point A). The mean percentage correct on the checklist after the completion of the School was 62.3% (Time Point B). There was also a statistically significant improvement between the completion of the School of POWHER (Time Point B), a mean of 62.3%, and continued clinical training for 6 months following completion of the School of POWHER with a mean of 71.0% (Time Point C). The largest amount of overall checklist improvement was seen before any clinical training occurred (Time Point A) when compared to completion of the School of POWHER (Time Point B). 

Specific improvement in each of the three prenatal skills categories was also statistically significant as seen in Table 5. In the category of history, taking the mean percentage correct on the checklist before any training occurred (Time Point A) was 26.3% compared to 58.8% at the completion of the School of POWHER (*p*-value = 0.005) (Time Point B). After six months of continued clinical training from the completion of the School of POWHER (Time Point C), the percent correct on the checklist improved to 87.1% (*p*-value < 0.0001). In the category of clinical skills, the mean percentage correct on the checklist before any training occurred (Time Point A) was 9.7% compared to 92.4% at the completion of the School of POWHER (*p*-value < 0.0001) (Time Point B). After six months of continued clinical training from the completion of the School of POWHER, the percent correct on the checklist was 81.4% (Time Point C). This decrease in overall percentage correct was not statistically significant (*p*-value = 0.19). In the category of counseling, the mean percentage correct on the checklist before any training occurred (Time Point A) was 27.7% compared to 86.2% at the completion of the School of POWHER (*p*-value < 0.0001) (Time Point B). After six months of continued clinical training from the completion of the School of POWHER (Time Point C), the percent correct in counseling on the checklist was 72.0%. This decrease in overall percentage correct was also not statistically significant (*p*-value = 0.09). 

The number of referrals increased over the study period although statistically insignificant. Before any clinical training occurred (Time Point A), only 17.8% of women were referred correctly. At the completion of the School of POWHER (Time Point B), appropriate referrals increased to 52.0% (*p* = 0.32). After six months of continued clinical training from the completion of the School of POWHER (Time Point C), appropriate referrals from the clinical checklist was 27.7%. The largest increase in referrals was seen in the category of clinical skills most commonly referred for the malposition of the fetus during late pregnancy. 

## 4. Discussion

When TBA training is successfully implemented in rural communities, TBAs increase their basic obstetric knowledge, are equipped for safe home deliveries, and are able to identify problems requiring a referral; factors which markedly improve obstetrical outcomes [19]. Recent systematic reviews have identified that successful programs are those that can be integrated into an existing healthcare system, continue skill development (monthly or bi-monthly) of its participants for an extended period of time, and provides them access to birth kits and resuscitation equipment [9,10,12,20].

Our study, in line with these systematic reviews, suggests that standardized and continuous clinical training improves the PCS of TBAs during home visits. Overall, our TBAs were more likely to provide more complete prenatal home visits after the completion of School of POWHER training. An improvement was seen consistently in each broad category (history, clinical skills, and counseling) of basic prenatal care. Continued improvement was seen after six months of post School of POWHER clinical training (10-months of overall clinical training). The finding of this present study are generally encouraging as other studies, including our own previous pilot study, have demonstrated that a lack of continuous training leads to a decrease in PCS [18]. This point is of utmost importance as many TBA programs in low-and-middle-income countries fail to provide follow-up training after the completion of their training program. We have demonstrated how PCS continue to improve, and the average amount of improvement expected to be seen within each broad category of prenatal care, when clinical training is structured and continuous. 

One of the biggest challenges that TBA training programs face is successfully measuring and reporting the outcomes of their didactic and clinical curriculum. We have previously published on the details of our 14-week School of POWHER training program [17]. With this present study, we focus on the standardization of our clinical curriculum and measuring its impact on PCS of our participant with a prenatal skills checklist. Our choice of standardization of clinical skills through a checklist was influenced by the success of other checklists in maternal healthcare [21,22,23]. The initial development of our prenatal skills checklist occurred from November 2016 through March 2017. Our own School of POWHER curriculum [17], WHO healthcare practices for birth attendants [15], and current guidelines for TBAs from the Guatemalan Ministry of Health [16] were consulted. By using this checklist to both standardize our clinical curriculum and measure PCS over time, we were able to report on a promising improvement of PCS during home visits. We believe our checklist had this effect via two ways: (1) our educators who trained the TBAs had an easy instrument that highlighted the key aspects of prenatal care from the clinical curriculum, (2) our TBAs had a simple and systematic approach during their prenatal home visits that reflected the curriculum learned during their training. Ultimately, the prenatal checklist standardized the clinical curriculum and successfully measured PCS improvement of each participant. 

Additionally, the results of our study demonstrated the referral capacities of our TBAs throughout their training. Our study participants saw a significant increase in appropriate referrals after the completion of the School of POWHER training program (Time Point B). Referrals decreased after six months of continuous, focused PCS training (Time Point C) but remained higher than referral capacities when TBAs had no training at all (Time Point A). It is likely that there was a decrease in referrals from Time Point B to Time Point C due to the strong emphasis on standardized PCS training during this six month time period. During this period, there was focused exposure and training on PCS where referral teaching by the clinical educators was not as structured. Furthermore, study observers reported that during time Point C, study participants verbalized that they were more comfortable waiting on certain referral points until the mother was more advanced in her pregnancy (for example maternal age or past medical history), thus, the study tool would not pick up this later referral as it was not captured during the observed visit. Despite this, even at Time Point B, when the most referrals were occurring by study participants, only 52% of women were accurately referred. Our study suggests that a major focus on PCS during continued training does not indicate an equivalent increase in referrals. Our results on referral capacities also demonstrate the need to have a structured teaching approach for referrals taught parallel to standardized PCS. 

This latter point is key as referrals must be timely and appropriate, which will only occur with access to properly trained birth attendants. Despite the push towards institutionalized births, the WHO has recognized that quality care during labor and delivery does not necessarily occur once a birth occurs in a hospital [24]. Specifically in Guatemala where there has been a push to birth in hospitals [4], untimely referrals during pregnancy by untrained healthcare personnel has inundated hospital waiting rooms leading to a minimal decrease in MMR. A possible solution to this problem lies in a new model of Birthing Homes (Casa Maternas) which have helped to reduce inappropriate referrals to the national hospitals in Guatemala [25]. This effective hybrid model between a home and institutional birth relies on the referral capacities of trained birth attendants that run the birthing homes. We propose that effective clinical training of TBAs not only affects the healthcare of women in rural settings but also has a larger impact on the burden of institutional births. 

Findings from our study should be interpreted within its limitations. Our sample size of 13 TBA participants was small. Nonetheless, our study was able to capture each participant before and after exposure to the School of POWHER training program and follow PCS outcomes over a considerable amount of time as the last data collection point was at eight and a half months of total clinical training. The small sample size potentially affected our statistical analysis of referrals performed by our TBA study participants, as the analytic sample at Time Point C is approximately 1/3 of the analytical sample at Time Point B and C. This difference in an already small sample size could mean that it is possible that there is not a sufficient sample size to detect subtle improvements in accurate referrals. Convenience sampling may introduce study bias but was unavoidable in the current study as it was extremely difficult to recruit and observe TBAs that were not participating in the School of POWHER training program. Additionally, participants knew that they were being observed and this could have unintentionally influenced their behavior during prenatal home visits. Participants might have been driven to practice the PCS they learned during the training program when the observer was present but this does not indicate that TBA participants were routinely implementing PCS during routine unobserved home visits.

Despite these limitations, the strength and originality of our study counteracts many of the biases put forth. Our standardization of prenatal clinical care and subsequent measurement of PCS prior to and after our training program via observation allows for a direct method to collect data that is superior to self-reporting.

## 5. Conclusions

This study highlights the feasibility of PCS improvement through a standardized and continuous clinical training program for TBAs. The prenatal checklist assessment tool also serves as an objective means to quantify TBA skills in order to evaluate and maintain their skills in low resource settings. The improvement of TBA prenatal clinical skills could benefit indigenous women in rural Guatemala, and other low-and middle-income countries that prefer and/or have no other option except home care during pregnancy and birth.

## Figures and Tables

**Table 1:** Traditional birth attendant participant demographics.

Characteristic	Average	Range
Age	40	22–54
Level of Education	2^nd^ grade	No formal education-8th grade (one outlier with one participant pursuing a technical degree in nutrition)
Marital Status	Married	1 single, 1 widowed, 11 married
Years of Experience	9	0–27
Dominant Language	Spanish, Tz’tujil, Kaqcuichel	

**Table 2:** The data collection characteristics.

Study Time Point	Dates of Data Collection	Total Weeks of Clinical Training	Total Weeks of Knowledge-Based Learning
Time Point A ^1^	March 22–April 7, 2017	0	0
Time Point B ^2^	June 30–August 16, 2017	14	14
Time Point C ^3^	November 7–December 12, 2017	38	14

^1^ Time point A occurred before any clinical instruction had begun; ^2^ Time point B occurred at the completion of 14-weeks of formalized POWHER curriculum; ^3^ Time point C occurred after six months of continuous clinical training from completion of the formalized POWHER curriculum.

**Table 3:** The variables included in the prenatal clinical skills observation checklist.

Variable	Definition	Reason for Referral
*History*		
Accurately calculates EDD	Using Naegele’s rule or pregnancy wheel.	If LMP unknown, referral for dating US.
Accurately calculates GA	Only if the mother knows LMP.	If LMP unknown, referral for dating US.
Age	Asks mother for an identification card if age is not known.	Referral recommended if AMA or less than 16 years old.
# of previous pregnancies	All pregnancies, including miscarriages.	
# of living children	All children; follows up if there is a discrepancy between total pregnancies and living children.	
Problems with previous pregnancies	Asks about prolonged labor, hemorrhage, problems with blood pressure, severe headache, fever during or after labor, infection during or after labor, prior C-sections.	Referral recommended if mother reports any prior problem. If C-section less than two years from current pregnancy recommends hospital birth.
Past medical history	Assesses any health conditions outside of pregnancy.	
Current medications	Assesses both OTC medications and street supplements.	
Documents history	Variables of importance are name, age, EDD, and prior issues of importance in order to report to the MOH. Not applicable for illiterate TBA.	
*Clinical skills*		
Washes hands	Uses soap and water or antiseptic solution.	
Measures blood pressure	Mother seated or supine, arm below the heart, places cuff 1–2 finger breadths above the elbow, places stethoscope in the area of the brachial artery under the cuff.	Refers if blood pressure greater than 140/90, or in “red zone” if illiterate TBA.
Measures heart rate	Locates radial pulse and measures for 60 s.	
Measures fundal height	Can use a measuring tape or hand measurements if TBA is illiterate.	Refers if there is a difference greater than 4 cm from fundal height compared to GA.
Listens to fetal heart rate	Can find fetal heart rate and distinguish from maternal heart rate and placental vessels.	Refers if fetal distress (FHR > 160 bpm, <100 bpm) persists throughout the visit.
Finds the position of fetus	Uses Leopold’s maneuvers.	If the fetal position is oblique, transverse, or breech, in late pregnancy, discusses with mother the dangers of home birth.
Documents all findings	Variables of importance are blood pressure findings to track trends over pregnancy, not applicable if illiterate.	
*Counseling*		
Discusses severe headaches	Explains to mom the risk of a severe headache and associated changes in vision.	Discusses with mother to report to the health post, local hospital, or private clinic.
Discusses severe abdominal pains	Demonstrates pain in the epigastric region.	Discusses with mother to report to the health post, local hospital, or private clinic.
Discusses vaginal bleeding	Explains the risk of spotting or bleeding at any point during the pregnancy.	Discusses with mother to report to the health post, local hospital, or private clinic.
Discusses fever	Explains chills and night sweats as symptoms of a fever.	Discusses with mother to report to the health post, local hospital, or private clinic.
Discusses location of birth	Discusses with mother and partner (if present) about the risks and benefits of both a home birth and a hospital birth).	
Discusses emergency plan	Discusses with mother and partner (if present) where to report and how to get there if an emergency arises during labor and delivery.	
Distributes prenatal vitamins	Hands a 30 day supply to mother.	
Counsels on the importance of prenatal vitamins	Explains the maternal and fetal benefit of prenatal vitamins to mother.	
Counsels on Td vaccine	Explains the vaccine schedule (1^st^ shot, 2^nd^ shot 2 months later, 3^rd^ shot months later) to mother and the importance of vaccination especially if a home birth is planned.	Refers to either health post or local hospital to received Td vaccine.

Abbreviations: EDD = estimated delivery date; GA = gestational age; LMP = last menstrual period; AMA = advanced maternal age; OTC = over the counter.

**Table 4:** The overall improvements in prenatal clinical skills.^1^

	Time Point A ^2^	Time Point B ^3^	Time Point C ^4^	*p*-value ^5^
Percentage of correctly performed prenatal clinical skills	25.8 (19.6)	62.3 (16.3)	71 (12.5)	Between A and B, 0.0001
				Between B and C, 0.034

^1^ Values entered as mean percentages (standard deviation); ^2^ Time point A occurred before any clinical instruction had begun; ^3^ Time point B occurred at the completion of 14-weeks of formalized POWHER curriculum; ^4^ Time point C occurred after six months of continuous clinical training from completion of the formalized POWHER curriculum; ^5^ Paired *t*-test.

**Table 5:** The specific checklist improvements per category ^1^.

Prenatal Skills	Time Point A ^2^	Time Point B ^3^	Time Point C ^4^	*p*-value ^5^
History (overall)	26.3%	58.8%	87.1%	Between A and B, 0.005
				Between B and C, <0.0001
Accurately calculates EDD	52.0%	82.0%	90.0%	-
Accurately calculates GA	7.5%	68.0%	90.0%	-
Age	36.0%	64.0%	92.0%	-
# of previous pregnancies	43.5%	64.0%	100.0%	-
# of living children	37.0%	64.0%	100.0%	-
Problems with previous pregnancies	35.5%	50.0%	100.0%	-
Past medical history	10.0%	52.0%	70.0%	-
Current medications	17.5%	44.0%	78.5%	-
Documents history	8.5%	100.0%	100.0%	-
Clinical skills (overall)	9.7%	92.4%	81.4%	Between A and B, ≤0.0001
				Between B and C, 0.19
Washes hands	0.0%	78.0%	45.0%	-
Measures blood pressure	0.0%	100.0%	93.0%	-
Measures heart rate	0.0%	94.0%	100.0%	-
Measures fundal height	0.0%	88.0%	93.0%	-
Listens to fetal heart rate	0.0%	100.0%	100.0%	-
Finds position of fetus	64.5%	94.0%	100.0%	-
Documents all findings	0%	100.0%	87.0%	-
Counseling (overall)	27.7%	86.2%	72.0%	Between A and B, ≤0.0001
				Between B and C, 0.09
Discusses severe headache	40.5%	89.0%	94.0%	-
Discusses severe abdominal pain	28.0%	89.0%	94.0%	-
Discusses vaginal bleeding	34.0%	89.0%	89.0%	-
Discusses fever	35.0%	89.0%	83.0%	-
Discusses location of birth	41.0%	86.0%	83.0%	-
Discusses emergency plan	43.5%	86.0%	84.0%	-
Distributes prenatal vitamins	0.0%	89.0%	78.0%	-
Counsels on importance of prenatal vitamins	0.0%	85.0%	65.0%	-
Counsels on Td vaccine	29.5%	89.0%	50.0%	-

^1^ Values entered as mean percentages; ^2^ Time point A occurred before any clinical instruction had begun; ^3^ Time point B occurred at the completion of 14-weeks of formalized POWHER curriculum; ^4^ Time point C occurred after six months of continuous clinical training from completion of the formalized POWHER curriculum; ^5^ Paired *t*-test.

## Supplementary Materials

The following are available online at http://www.mdpi.com/2227-9032/6/2/60/s1, Figure S1: The School of POWHER curricular overview, Figure S2: Prenatal Clinical Assessment Tool.
