# Supplementary material for: Impact of Standardized Prenatal Clinical Training for Traditional Birth Attendants in Rural Guatemala
Source: Healthcare (Basel). 2018 Jun 9;6(2):60. doi: 10.3390/healthcare6020060 (PMC6023520; doi:10.3390/healthcare6020060)
Supplement: Supplementary file 1 [file healthcare-06-00060-s001.pdf]

Healthcare **2018**, *6*, 60; doi:10.3390/healthcare6020060

Supplementary Materials: Impact of standardized prenatal clinical training for traditional birth attendants in rural Guatemala

Hernandez Sasha, Oliveira Jessica, Jones Leah, Chumil Juan, Shirazian Taraneh

**Week 1**	Day 1: An Overview of Maternal-Infant Care
	Day 2: The Role of the Birth Attendant, Health Care Resources and the Emergency Plan
**Week 2**	Day 3: Sexual Organs, Sexual Desire, and Menstruation, Ovulation, and Fertilization
	Day 4: The Natural Development of Pregnancy
**Week 3**	Day 5: The Choice of Motherhood, The Importance of Prenatal Care- Part 1
	Day 6: The Importance of Prenatal Care-Part 2
**Week 4**	Day 7: Pregnancy Complications, Part 1
	Day 8: Pregnancy Complications, Part 2
**Week 5**	Day 9: Signs and Symptoms of Danger during Pregnancy
	Day 10: Case Studies on Prenatal Care, Exam 1
**Week 6**	Day 11: Review Exam 1
	Day 12: Tetanus Vaccine, Introduction to Stages of Labor
**Week 7**	Day 13: Uncomplicated Labor: Stage One and Two
	Day 14: Uncomplicated Labor: Stage Two and Three
**Week 8**	Day 15: Review of Stages of Labor, Basic Care of the Newborn
	Day 16: Complications during Labor and Delivery
**Week 9**	Day 17: When to Refer During Labor
	Day 18: Neonatal Resuscitation, Immediate Post-Partum Care, Maternal Lactation
**Week 10**	Day 19: Maternal Lactation Review, Post-Partum Care: Day 1 & 4
	Day 20: Review of Normal and Complicated Labor, Exam 2
**Week 11**	Day 21: Medicinal Plants, Sharing of Experiences during Labor
	Day 22: Infection Prevention and Management, Sterilization of Birth Tools
**Week 12**	Day 23: Family Planning Methods
	Day 24: Case Studies: Family Planning, Self Breast Exam
**Week 13**	Day 25: Vaginal Infections and STIs
	Day 26: Nutrition and Malnutrition
**Week 14**	Day 27: Complicated Labor
	Day 28: Cumulative review, Final Exam

Figure S1. The School of POWHER curricular overview

	**HISTORY**	
	**Task**	**Performed (✓/ ✗/NA)**
1	If mother knows LMP, accurately calculates EDD	
2	If mother knows LMP, calculates weeks of gestation	
3	Focused OB History:	
	Age	
	# of previous pregnancies	
	# of living children	
	Asks about problems with previous pregnancies	
4	Refers appropriately if prior complicated pregnancy	
5	Asks about any significant past medical history	
6	Asks about current medications	
7	Documents history	
	**CLINICAL SKILLS**	
	**Task**	**Performed (✓/ ✗/NA)**
1	Washes hands with soap and water or uses anti-septic	
2	Accurately measures blood pressure	
3	Accurately measures heart rate	
4	Correctly measures fundal height	
5	Listens to fetal heart rate with Doppler	
6	Finds position of fetus	
7	If fetal position is oblique, transverse, or breech, discusses with mother appropriate places to birth	
8	Documents all findings	
	**COUNSELING**	
	**Task**	**Performed (✓/ ✗/NA)**
1	Reviews signs and symptoms of danger:	
	Severe headache	
	Abdominal pain	
	Vaginal bleeding	
	Fever	
2	If mother reports any warning signs, appropriately refers	
3	Discusses labor plan with mother:	
	Asks where the birth will take place	
	Asks what the mother plans to do in case of an emergency	
4	Distributes pre-natal vitamins	
5	Counsels mother on importance of pre-natal vitamins	
6	If mother does not have Td vaccine, counsels on vaccination before delivery	

Figure S2. Prenatal Clinical Assessment Tool

© 2018 by the authors. Licensee MDPI, Basel, Switzerland. This article is an open access article distributed under the terms and conditions of the Creative Commons Attribution (CC BY) license (http://creativecommons.org/licenses/by/4.0/).
